# The Role of the Gustatory System in the Coordination of Feeding

**DOI:** 10.1523/ENEURO.0324-17.2017

**Published:** 2017-11-20

**Authors:** Vladimiros Thoma, Kimiko Kobayashi, Hiromu Tanimoto

**Affiliations:** 1 Department of Biology, Miyagi University of Education, Sendai, Miyagi 980-0845, Japan; 2 Graduate School of Life Sciences, Tohoku University, Sendai, Miyagi 980-8577, Japan

**Keywords:** coordination, evolution, feeding behavior, gustatory sensory neuron, neuroethology, taste

## Abstract

To survive, all animals must find, inspect, and ingest food. Behavioral coordination and control of feeding is therefore a challenge that animals must face. Here, we focus on how the gustatory system guides the precise execution of behavioral sequences that promote ingestion and suppresses competing behaviors. We summarize principles learnt from *Drosophila*, where underlying sensory neuronal mechanisms are illustrated in great detail. Moreover, we compare these principles with findings in other animals, where such coordination plays prominent roles. These examples suggest that the use of gustatory information for feeding coordination has an ancient origin and is prevalent throughout the animal kingdom.

## Visual Abstract

**Figure:**
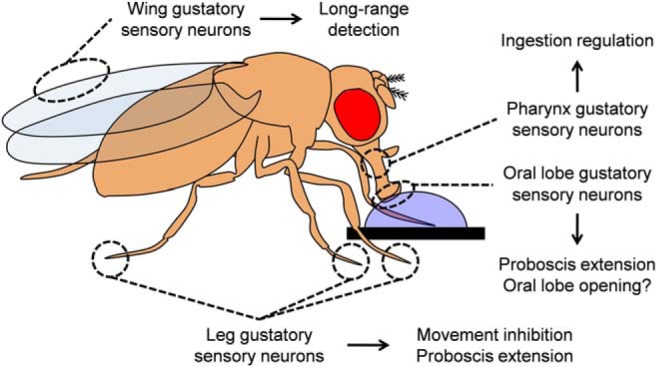

## Significance Statement

Efficient feeding requires coordination. We synthesize findings across diverse species, highlighting the widespread and ancient role of taste in feeding coordination.

## Introduction

All living organisms need energy to survive and reproduce. In contrast to plants and fungi, which rely on photosynthesis and absorption respectively, animals acquire energy by feeding on other organisms. As feeding behavior is essential for survival, animals have evolved specialized feeding structures such as mouths, teeth, tongues, and proboscises for food ingestion. These structures underlie equally diverse ingestion strategies. For example, many animals use their mouths to physically break down their food, others swallow their prey whole, and some feed by sucking nutritious fluids like nectar. Despite this diversity, food ingestion follows three general principles. (1) First, the animal must decide whether or not to accept a potential meal. (2) Upon acceptance, the animal must precisely execute behavioral sequences such as synchronized use of different effectors like jaws and tongues. (3) At the same time, competing behaviors like locomotion are often suppressed, so that the animal can focus on feeding. These two latter features ensure coordinated, efficient ingestion.

Sensory information, and especially taste, play critical roles in these three steps. Here, we focus on the gustatory control of steps (2) and (3), directing the reader to other reviews as to step (1) (Scott, 2005; Yarmolinsky et al., 2009; Liman et al., 2014; Joseph and Carlson, 2015). In his seminal review, published more than two decades ago, Reinhard Stocker provided a detailed description of the anatomy and physiology of the chemosensory system of *Drosophila* (Stocker, 1994). Based on these data, he hypothesized that the gustatory organs distributed throughout the body of the fruit fly have specialized functions. This idea has been remarkably substantiated by recent progress, which highlights the roles of distinct gustatory sensory neurons in regulating different aspects of feeding. Here, we review this progress and discuss the gustatory sensory mechanisms that underlie feeding coordination in other animals. By comparing the gustatory neuroethology of feeding in different animals, this review tries to extract common principles and discuss when these conserved systems evolved.

## Execution of Behavioral Sequences during Feeding

To ingest food, humans and other primates use their hands to deliver it to their mouth (McGraw and Daegling, 2012). Consequently, chewing and secretion of saliva ensures that food is broken down physically and chemically. Once this is achieved, swallowing passes the food toward the alimentary canal for further digestion. Eventually, animals reach satiation and stop ingesting food. Similar behavioral sequences characterize the ingestion behaviors of many animals. Indeed, even “primitive” animals like jellyfish have a stereotyped sequence of feeding (Lindstedt, 1971b). Despite the prevalence of such sequences, the underlying sensory neuronal mechanisms that ensure their precise execution are not well understood.

Studies in insects provided important early contributions toward understanding the gustatory sensory control of feeding sequences. Unlike mammals, where the tongue is the main taste organ, insects have broadly distributed gustatory organs that include the legs, wings, genitalia, the surface and interior of the proboscis and, in some species, the antennae and maxillary palps (Stocker, 1994). In his influential book, Vincent Dethier provided detailed descriptions and studies of blowfly feeding behavior (Dethier, 1976). From a distance, a hungry blowfly relies on visual and olfactory cues to guide it toward potential food sources. When the blowfly eventually steps on food, it immediately stops, orients itself toward the food and extends its proboscis. Consequently, it opens its oral lobes (labella), located at the tip of the proboscis, and begins sucking food until it is satiated. Finally, the fly retracts its proboscis and eventually moves away, ignoring further encounters with food. When specific gustatory organs are stimulated, blowflies typically execute different subsets of feeding behavior (Pollack, 1977). Based on such observations, Dethier concluded that the legs control proboscis extension, while the gustatory hairs and gustatory pegs of the proboscis control the opening of its lobes and the sucking of food, respectively (Dethier, 1976). Chapman extended this observation to other insects (Chapman, 1995). Moreover, he argued that feeding sequences are not fixed, partly because gustatory organs early in the sequence can be overridden by more “downstream” organs. For example, grasshopper nymphs will lift their legs to avoid contact with a deterrent-covered leaf while eating it (White and Chapman, 1990). In blowflies, leg-driven proboscis extension is suppressed if an aversive substance is presented on the proboscis (Dethier et al., 1956). Despite their importance, these early studies did not have the resolution of individual neurons, leaving underlying neuronal mechanisms unanswered.

**Figure 1.**
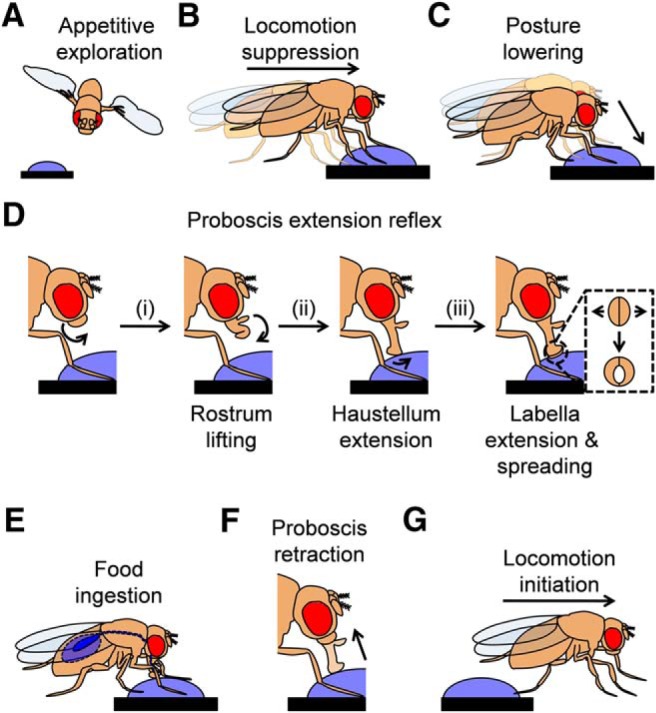
The feeding sequence of *Drosophila*. ***A***, Initially, a hungry fly will search for food. This appetitive exploration relies on visual and chemical cues. As soon as the fly steps on food, it suppresses its movement (***B***), lowers its posture (***C***), and extends its proboscis (***D***). The extension of the proboscis comprises of four steps that use different muscles (i-iii). A schematic of the surface of the proboscis, showing the spreading of the labella from below, is shown in the last step (inset). Consequently, (***E***) the fly ingests food until satiated and then (***F***) retracts its proboscis and (***G***) moves away.

**Figure 2.**
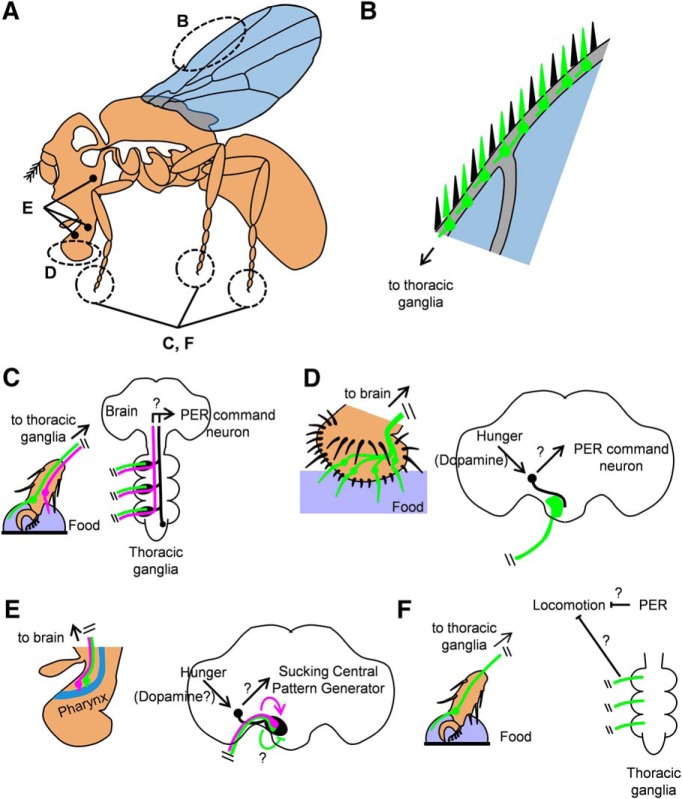
Neuronal circuits coordinating feeding in *Drosophila*. ***A***, Schematic of an adult fruit fly, indicating the central nervous system, comprising of the brain and thoracic ganglia (white). The position of the gustatory organs (wings, tarsi, labella, and pharyngeal organs) are highlighted. ***B***, GRNs in the wings (green) are critical for long-range foraging. ***C***, Circuits controlling the PER on leg stimulation. Out of two groups of leg GRNs, brain-projecting GRNs (magenta) are more critical for PER, likely through more direct interactions with a brain command neuron. Locally-projecting GRNs (green) exert a smaller role, likely through SGNs (black). ***D***, Circuits controlling PER on labelar stimulation. Labelar GRNs (green) connect to SGNs in the brain (black) that integrate taste and hunger. ***E***, Circuits controlling food ingestion. Pharyngeal GRNs regulate feeding by prolonging (magenta cell) or decreasing (green cells) ingestion time. The sucking command neuron (black) directly receives information from pharyngeal GRNs, integrates it with hunger and likely activates the central pattern generator that controls pumping. ***F***, Circuits controlling locomotion suppression when feeding. Locally-projecting leg GRNs (green) suppress movement on food encounter. Proboscis extension also suppresses locomotion, although the underlying circuits are unknown. Question marks (?) indicate speculative or unknown pathways. For simplicity, only one or a few GRNs or SGNs per category are shown.

The advent of *Drosophila* neurogenetics has enabled targeted manipulation of neurons, allowing experiments that directly address their functions. Fruit flies and blowflies share a similar feeding sequence (Fig. 1; Pool and Scott, 2014) and gustatory system (Fig. 2*A*). Typically, the detection of food causes the aggregation of many fruit flies on it, both in nature and in the laboratory. Surprisingly, this aggregation is dramatically reduced when the gustatory receptor neurons (GRNs) in the wings (Fig. 2*B*, green) are specifically eliminated (Raad et al., 2016). The authors of this study propose that GRNs in the wings detect non-volatile chemicals in microdroplets, which are produced by flying over liquids, and can therefore facilitate appetitive exploration (Fig. 1*A*). Once the flies land on food, additional gustatory organs can be stimulated. Like in blowflies, stimulating GRNs in the legs of hungry fruit flies with food (Fig. 2*C*) elicits the proboscis extension reflex (PER; Fig. 1*D*). PER can also occur after stimulation of the labelar gustatory hairs (Fig. 2*D*; Shiraiwa and Carlson, 2007). Spontaneous proboscis responses have also been reported (Fujita and Tanimura, 2011), but they are very infrequent (Mann et al., 2013) and less likely to proceed to full extension in wild-type flies (Chabaud et al., 2006). Such responses may be of lesser importance under normal circumstances, as the legs typically contact the food first. Leg-driven PER is controlled by a small group of highly sensitive sugar GRNs, located in the tip of each leg (Fig. 2*C*, magenta; Miyamoto et al., 2013; Ling et al., 2014; Thoma et al., 2016). They are likely to constantly evaluate potential food during walking. Moreover, they project directly to the brain, ensuring rapid proboscis extension as soon as food is detected. Such anatomical and physiological properties likely evolved to support a specialized and crucial role in the initiation of feeding.

Detailed examination of the PER reveals that it can be broken down to four sequential steps, namely lifting of the rostrum, extension of the haustellum and extension and spreading of the labella (Fig. 1*D*). These steps are independent: later steps can be induced without earlier steps (Schwarz et al., 2017), showing that the effectors within the proboscis are not organized as a chain reaction, but rather centrally coded via “command” interneurons (Flood et al., 2013). Interestingly, at least one step, the spreading of the labella (Fig. 1*D*, inset), was elicited more robustly by labelar rather than tarsal stimulation, in line with findings in the blowfly (Pollack, 1977). Such differences in effectiveness may enable fine-tuning of the feeding response based on positional information, in the case of a highly localized appetitive stimulus.

After the proboscis is extended, flies start to ingest food (Fig. 1*E*), bringing it into contact with pharyngeal GRNs (Fig. 2*E*), which are in close proximity with the central pattern generator that generates sucking behavior (Manzo et al., 2012). Pharyngeal GRNs regulate food consumption by influencing the duration of ingestion (LeDue et al., 2015; Joseph et al., 2017). Surprisingly, two populations of sugar-sensitive pharyngeal GRNs have opposite effects on consumption: a group of gustatory receptor-expressing cells promotes it (LeDue et al., 2015), while a pair of ionotropic receptor-expressing cells inhibits it (Fig. 2*E*, magenta and green, respectively; Joseph et al., 2017). The ionotropic receptor-expressing cells were proposed to prevent overconsumption following prolonged ingestion, because their calcium responses are slower than those of the gustatory receptor-expressing cells (Joseph et al., 2017). Interestingly, following the ingestion of a small amount of sugar solution, flies execute a local appetitive search, a behavior also controlled by pharyngeal GRNs (Murata et al., 2017). Taken together, these findings emphasize the role of highly specialized GRNs in the execution of the *Drosophila* feeding sequence and in the precise control of each step.

Because *Drosophila* feeding responses are not simple monosynaptic reflexes (Gordon and Scott, 2009), the specialized functions of GRNs are likely enacted by central neurons. Recent studies have identified several second-order gustatory neurons (SGNs) that receive direct input from GRNs and control different aspects of feeding (Fig. 2*C-E*; Kain and Dahanukar, 2015; Miyazaki et al., 2015; Yapici et al., 2016; Kim et al., 2017). For example, two sets of SGNs in the thoracic ganglia receive input from sugar GRNs in the legs and their activation is sufficient, but not necessary, to elicit PER (Fig. 2*C*, black; Kim et al., 2017). This suggests redundancy in the neuronal pathways controlling this behavior, in line with findings about the corresponding leg GRNs (Thoma et al., 2016). Interestingly, two different groups of brain SGNs were recently shown to control distinct aspects of the feeding sequence. The first group receives input from labelar GRNs and controls PER (Fig. 2*D*, black; Kain and Dahanukar, 2015), while the second group receives input from pharyngeal GRNs and controls ingestion (Fig. 2*E*, black; Yapici et al., 2016). Both groups not only enact the functions of their associated GRNs, but also integrate gustatory information with starvation state, the latter likely being conveyed to them by dopaminergic circuits (Inagaki et al., 2012; Marella et al., 2012). Future studies will be useful to place additional feeding interneurons (Flood et al., 2013; Mann et al., 2013; Pool et al., 2014) in the broader context of these established gustatory neuronal circuits.

Although gustatory information is critical for feeding, nutritional needs and the caloric content of food also play important roles. Nutritional needs are coded by central neurons, which determine the substances to be ingested (Bjordal et al., 2014; Jourjine et al., 2016). Importantly, because nutrients can reach the brain within seconds of feeding initiation (Itskov et al., 2014), caloric information can be used in real-time to regulate ingestion (Qi et al., 2015). This is accomplished by brain neurons, some of which express gustatory receptors (Miyamoto et al., 2012; Dus et al., 2013). For example, brain neurons expressing a fructose receptor detect this sugar in the hemolymph, and are involved in ingestion regulation and appetitive learning (Miyamoto et al., 2012). The presence of nutrient-detecting pathways is likely advantageous to flies, as some naturally occurring sugars are sweet but offer no nutrition. These findings demonstrate that there are complex interactions between taste, hunger, caloric content and nutritional needs, and they are crucial for the tight regulation of feeding.

## Feeding Sequences of Other Animals

**Figure 3.**
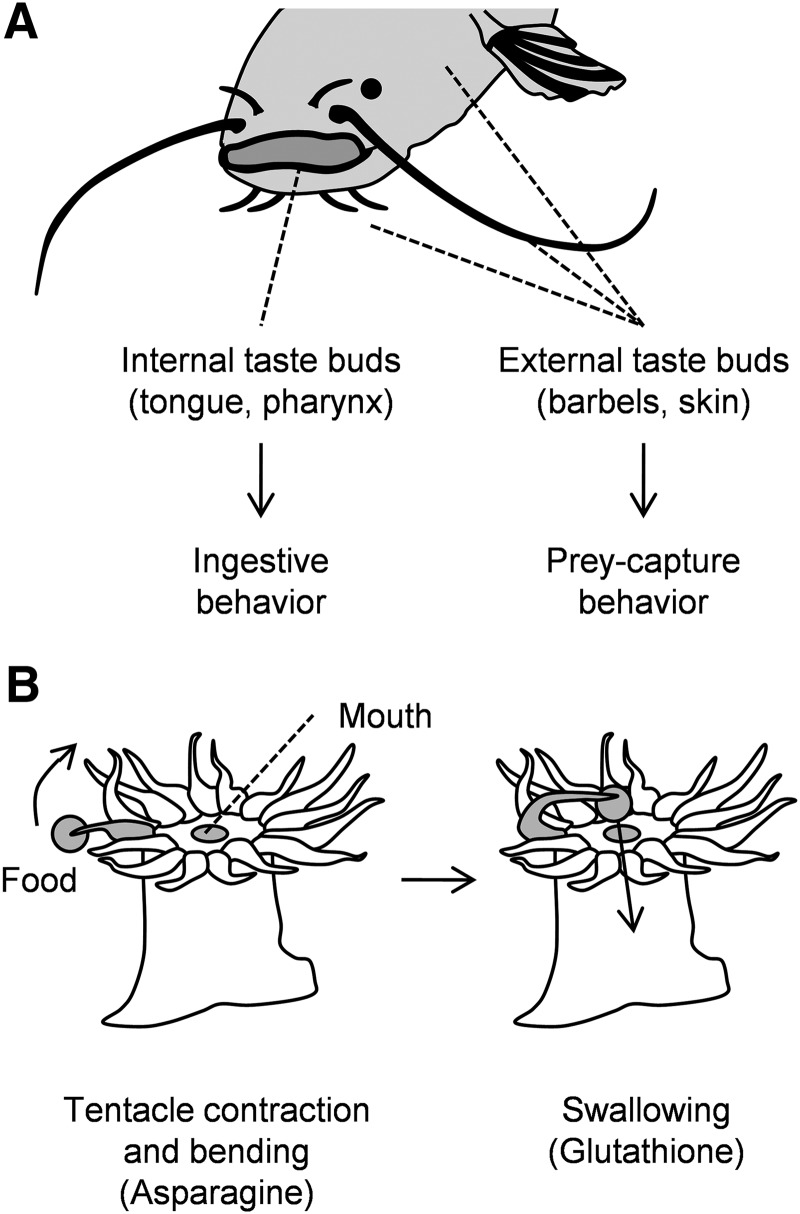
The role of gustatory information in the feeding coordination of catfish and sea anemones. ***A***, Catfish have external and internal taste buds. The former control prey-capture behaviors, while the latter control ingestive behaviors such as chewing and swallowing. ***B***, The sea anemone *Anthopleura* captures food (gray circle) with its tentacles, brings it to its mouth, and swallows it. Asparagine and glutathione, likely released from sting-injured prey, control tentacle movements and swallowing, respectively.

Like insects, many aquatic animals have taste sensors that are widely distributed over their body. In several cases, these have been shown to play different roles in feeding behavior. Catfish provide a striking example. They have taste buds not only in their mouth and pharynx, but also on their entire body surface, and are often described as “swimming tongues” (Caprio et al., 1993). Their surface taste buds are involved in capturing prey with their mouth, while internal taste buds control chewing and swallowing (Fig. 3*A*; Atema, 1971). Other aquatic animals also have multiple taste organs. In blue crabs and leeches engaged in feeding, the rejection of aversive tastants is controlled by internal, and not external, taste sensors (Kornreich and Kleinhaus, 1999; Aggio et al., 2012). In lobsters, taste sensors in the legs are required for a food-clasping response (Borroni et al., 1986). Although the functions of taste organs are not always clear, these examples show that they are often specialized.

Jellyfish and anemones belong to the phylum *Cnidaria*, which represents one of the most basal animal phyla with nervous systems. They typically capture prey with sting-equipped tentacles and consequently transfer it to the mouth for swallowing and digestion. In the freshwater polyp *Hydra*, tentacle retraction and mouth opening are triggered by the small peptide glutathione, presumably released from sting-injured prey (Loomis, 1955). After swallowing, the mouth constricts to form a “neck” that prevents prey escape, a behavior controlled by tyrosine, which is detected inside the mouth (Blanquet and Lenhoff, 1968). In the sea anemone *Anthopleura*, tentacle bending and ingestion are controlled by asparagine and glutathione, respectively (Fig. 3*B*; Lindstedt, 1971a). In the jellyfish *Aglantha*, orientation of the mouth toward tentacle-captured prey and engulfing lip movements, which promote ingestion, are controlled by distinct conduction pathways (Mackie et al., 2003), although gustatory signals that activate these pathways are unknown. These examples suggest that coordination of feeding through multiple gustatory sensors has ancient origins.

How is feeding coordinated in animals with centralized taste systems, like mammals? In mice, starvation alters the activity of hypothalamic circuits, which causes animals to forage for food. The sensory detection of food resets activity in these circuits, inhibiting foraging and facilitating the transition to ingestive behaviors (Chen et al., 2015). However, the specific role of taste information in these circuits was not investigated in detail. The initiation of ingestion, namely food delivery to the mouth, must rely on other sensory modalities, as mammals lack external taste sensors. However, taste sensory cells in the mouth may play different roles in later phases of ingestion. For example, like in fruit flies, pharyngeal taste sensors in mammals may be important for swallowing (Yapici et al., 2016). Spector and Glendinning suggest that the vertebrate taste system serves three functions: stimulus identification, ingestive motivation and digestive preparation. They speculate that different taste afferent inputs control these different functions (Spector and Glendinning, 2009). Future research is awaited to examine this hypothesis.

## Suppression of Competing Behaviors during Feeding

Feeding is typically incompatible with other behaviors. For example, when feeding on blood, leeches can endure aversive chemicals applied to the animal’s chemosensory organ (Kornreich and Kleinhaus, 1999) and electric shocks (Gaudry et al., 2010). Strikingly, highly dissected leeches lacking a body wall and internal organs will still initiate and commit to feeding, showing that they can even ignore severe trauma to obtain a meal (Gaudry and Kristan, 2009). Such remarkable concentration on feeding is observed in many other animals including jellyfish (Mackie et al., 2003), crayfish (Krasne and Lee, 1988), mollusks (Davis et al., 1977; Advokat, 1980; Kovac and Davis, 1980; Norekian and Satterlie, 1996), and insects (Mann et al., 2013; Thoma et al., 2016). Despite the widespread occurrence of this phenomenon, the underlying sensory mechanisms are only partially understood.

In his classical studies, Dethier observed that hungry blowflies, upon encountering food, will immediately stop walking (Dethier, 1976). Because the initial food contact must be made with the leg, before other gustatory organs are stimulated, leg GRNs likely control this behavior. The specific gustatory mechanisms underlying food-induced locomotion suppression have been recently elucidated in *Drosophila* (Mann et al., 2013; Thoma et al., 2016). Fruit flies have two anatomically distinct sugar GRNs in the legs: some project directly to the brain, whereas the rest project locally to the thoracic ganglia (Fig. 2*C*, magenta and green, respectively). The locally projecting neurons are specifically required for locomotion suppression (Thoma et al., 2016), most likely by influencing the motor neurons controlling leg movements, which are also located in the thoracic ganglia (Fig. 2*F*, green; Tuthill and Wilson, 2016). Moreover, when the fruit fly starts to feed, the extension of the proboscis itself inhibits locomotion via as yet unidentified circuits (Mann et al., 2013). Therefore, two dedicated neuronal mechanisms ensure that locomotion is inhibited not only as an initial response to food, but also throughout ingestion.

In the *Drosophila* larva, activation of a small set of interneurons expressing the neuropeptide hugin suppresses feeding and initiates locomotion, providing the basis for the mutual exclusivity of these competing behaviors (Schoofs et al., 2014). The dendrites of the hugin-expressing neurons are in close proximity to the GRN axon terminals, and their axons project to pharyngeal muscles (Melcher and Pankratz, 2005). This strongly suggests that gustatory information, by directly acting on hugin neurons, is critical for choosing whether to feed or move in the larva. Taken together, these results highlight *Drosophila* as a well-established model system for understanding the gustatory and neuronal mechanisms that govern the behavioral switch towards feeding.

**Figure 4.**
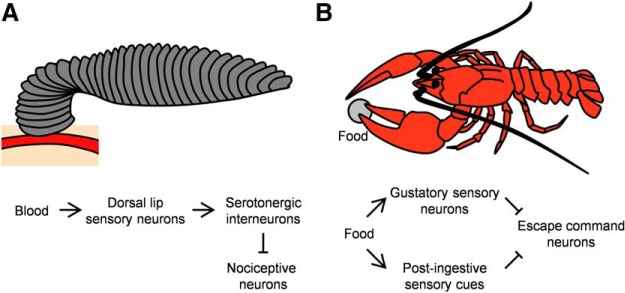
Neuronal circuits underlying suppression of escape responses during feeding in leeches and crayfish. ***A***, In leeches, stimulation of taste sensory neurons in the lip inhibits nociceptive neurons through serotonergic interneurons. ***B***, In crayfish, taste and postingestive information suppresses the escape command neurons.

Many other animals can suppress competing behaviors when feeding. In crayfish, mollusks and leeches, painful stimuli elicit defensive responses such as escape, but these are suppressed in the presence of food or when feeding (Davis et al., 1977; Advokat, 1980; Kovac and Davis, 1980; Krasne and Lee, 1988; Gaudry and Kristan, 2009). Behavioral and electrophysiological studies highlight two opposing mechanisms that underlie this suppression (Fig. 4). In leeches, blood stimulates chemosensory neurons in the lip, which in turn inhibit the nociceptive neurons, likely through serotonergic interneurons. The escape command neurons remain unaffected (Fig. 4*A*; Gaudry and Kristan, 2009, 2012). In contrast, in crayfish and in mollusks, food and postingestive stimuli inhibit the escape command neurons (Fig. 4*B*; Davis et al., 1977; Kovac and Davis, 1980; Krasne and Lee, 1988). These opposing mechanisms may underlie different food requirements or feeding habits. Such circuits should be able to “weigh” appetitive and aversive stimuli in the context of the animal’s hunger state, and carry out the cost-benefit analysis necessary for adaptive behavior.

## Conclusions

Feeding behavior is essential for all animals and it has played a critical role in shaping their evolution. Nervous systems may have evolved to facilitate more efficient feeding (Arendt et al., 2015), predation or predation avoidance (Kristan, 2016). Because chemosensation has ancient origins that trace back to bacteria (Baluska and Mancuso, 2009), taste sensory neurons were most likely important for improving feeding efficiency from an early stage. The examples provided here highlight the important role of taste in coordinating feeding in several animal species. However, our understanding of feeding neuroethology still remains limited to a handful of organisms. To understand the evolution of the neural circuits underlying feeding, we must first expand our knowledge to a broader set of animals that better captures the full diversity of the animal kingdom.

## Synthesis

Reviewing Editor: Thomas McHugh, RIKEN Brain Science Institute Decisions are customarily a result of the Reviewing Editor and the peer reviewers coming together and discussing their recommendations until a consensus is reached. When revisions are invited, a fact-based synthesis statement explaining their decision and outlining what is needed to prepare a revision will be listed below. The following reviewer(s) agreed to reveal their identity: Nilay Yapici, Hokto Kazama.

Summary

This manuscript, “The role of the gustatory system in the coordination of feeding”, summarizes how gustatory neurons control the behavioral sequences of feeding, as well as suppress other behaviors during food intake. It begins by introducing classic studies on blowflies and then explains how genetic tools available in Drosophila have allowed deeper understanding of neural mechanisms underlying feeding behavior. Further, it includes comparisons with other animals and ends by describing the competition between feeding and other behaviors.

Both referees have found the review to be timely and well written and only have minor suggestions for its improvement. Their comments included, “The manuscript is well written, comprehensive and describes the gustatory/feeding field in flies very clearly. The citations cover the old and the new literature and very appropriately chosen” and “The text was easy to read and I have only a few minor comments to add”.

Minor points to address:

1. l.84. “...or even spontaneously, as flies where external GRNs are transformed to mechanosensors can still ingest food (LeDue et al., 2015).” Using the result of LeDue et al. on poxn mutant to support this notion is a bit indirect. References reporting the observation of spontaneous PER in wild type animals can be added.

2. l.116. “Recent studies have identified several second-order gustatory neurons (SGNs)...”. Although the study did not test the functional aspects, Miyazaki et al., J Neurogenetics (2015) should be cited when SGNs are introduced.

3. Please correct the grammatical errors in lines 135 and 149.

4. On additional comment from Reviewer #1 contained a suggestion to include nutrient sensing mechanism in flies and state the fact that gustatory systems, hunger sensation and nutrient sensing as a whole control feeding behavior in flies and in other organisms. While optional, the reviewer felt this may improve an already well written manuscript.

## Author Response

We thank the reviewers for their encouraging words and constructive criticisms. Additionally, we thank the editor for quick and efficient handling of the paper. We have now revised the manuscript to address the issues raised during the review process. New text is labelled in the manuscript with a blue font color, while deletions are marked with strikethrough. Please find our point-by-point response to the issues raised below.

Minor points to address:

1. l.84. “...or even spontaneously, as flies where external GRNs are transformed to mechanosensors can still ingest food (LeDue et al., 2015).” Using the result of LeDue et al. on poxn mutant to support this notion is a bit indirect. References reporting the observation of spontaneous PER in wild type animals can be added.

We fully agree with the reviewer and are now making this point without a reference to the poxn data (lines 85-88). Instead, we cite Mann et al., 2013, Neuron and Chabaud et al., 2006, J Comp Physiol A Neuroethol Sens Neural Behav Physiol. Both these papers directly measured spontaneous PER of Drosophila, in wild-type and control genotypes.They found that spontaneous proboscis movements do occur, but they are infrequent and do not usually proceed to full extension (Lines 85-88).

2. l.116. “Recent studies have identified several second-order gustatory neurons (SGNs)...”. Although the study did not test the functional aspects, Miyazaki et al., J Neurogenetics (2015) should be cited when SGNs are introduced.

We have now added the reference to the work by Miyazaki et al. (Line 124).

3. Please correct the grammatical errors in lines 135 and 149.

We apologize for these errors and have now corrected them (line 153 and line 167).

4. On additional comment from Reviewer #1 contained a suggestion to include nutrient sensing mechanism in flies and state the fact that gustatory systems, hunger sensation and nutrient sensing as a whole control feeding behavior in flies and in other organisms. While optional, the reviewer felt this may improve an already well written manuscript.

The reviewer's point is well taken. Feeding regulation by hunger, nutritional needs and caloric content of food is a fascinating topic in itself that could easily be the focus of the entire review article. Although we chose to focus on the gustatory mechanisms controlling feeding, we fully acknowledge that taste, hunger and nutrition all interact to regulate feeding. Therefore, we now include a succinct summary and discussion of key findings in the field (lines 138-150).

Additional comments:

1. A recently published study by the lab of Prof. Tanimura (Murata et al. 2017) came to our attention during the revision. This study describes a new appetitive behaviour - local appetitive search following ingestion of a small amount of sweet solution - and shows that it is controlled by sweet taste sensory neurons in the pharynx. Because of its interest and relevance, we have updated our manuscript with a brief description of the study (lines 116-118).

2. We have changed the configuration of Figure 3 (horizontal to vertical) to make it easier to place in a single column in the final document.

3. We now include a Visual Abstract.
